# The dynamic communities of oral microbiome in neonates

**DOI:** 10.3389/fmicb.2022.1052525

**Published:** 2022-12-06

**Authors:** Haiying Guo, Jin Li, Hantao Yao, Yina Liu, Yaoting Ji, Jing Zhang, Yun Zhao, Minquan Du

**Affiliations:** ^1^The State Key Laboratory Breeding Base of Basic Science of Stomatology (Hubei-MOST) & Key Laboratory of Oral Biomedicine Ministry of Education, School & Hospital of Stomatology, Wuhan University, Wuhan, Hubei, China; ^2^Department of Stomatology, The First Affiliated Hospital of Zhengzhou University, Zhengzhou, Henan, China; ^3^Department of Oral Medicine, Xiangya Hospital, Central South University, Changsha, Hunan, China; ^4^Maternal and Child Hospital of Hubei Province, Tongji Medical College, Huazhong University of Science and Technology, Wuhan, Hubei, China

**Keywords:** dynamics, oral microbiome, neonates, maternal microbiome, high-throughput sequencing

## Abstract

The oral microbiome, associated with both oral disease and systemic disease, is in dynamic status along the whole life, and many factors including maternal microbiomes could impact the oral microbiome. While fewer studies have been conducted to study the characteristics of the oral microbiome in neonates and the associated maternal factors. Hence, we collected the microbiome of 15 mother-infant pairs across multiple body sites from birth up to 4 days postpartum and used high-throughput sequencing to characterize the microbiomes in mothers and their neonates. The oral microbiome in the neonates changed obviously during the 4 days after birth. Many bacteria originating from the vagina, skin, and environment disappeared in oral cavity over time, such as *Prevotella bivia* and *Prevotella jejuni*. Meanwhile, *Staphylococcus epidermidis RP62A phage SP-beta*, predominate bacterium in maternal skin microbiome and *Streptococcus unclassified*, main bacterium in vaginal microbiome, obviously increased in neonatal oral microbiome as time went on. Interestingly, as time progressed, the composition of the oral microbiome in the neonates was more similar to that of the milk microbiome in their mothers. Moreover, we found that the changes in the predominant bacteria in the neonates were in line with those in the neonates exposed to the environment. Together, these data described the sharp dynamics of the oral microbiome in neonates and the importance of maternal efforts in the development of the neonatal microbiome.

## Introduction

The oral microbiome, ranking the second in the microbiomes of the human body, plays a key role in both oral and systemic diseases, such as caries, periodontitis, preterm low birth weight, Alzheimer’s disease, and depression (Agnello et al., 2017; Zhang et al., 2018; Deo and Deshmukh, 2019; Guo et al., 2021; Wingfield et al., 2021; Ye et al., 2022). As well known, the oral microbiome is in a dynamic balanced state and many factors including the host gene, delivery mode, breastfeeding habits, antibiotics, environment, and physiological changes could influence its composition (Xu et al., 2015; Tamburini et al., 2016; Kaan et al., 2021). Among these factors, the effects of host gene, delivery mode, antibiotics used in pregnancy and lactation, and breastfeeding habits on the composition of microbiome are essentially the influences of the maternal microbiomes on the neonatal microbiomes (Dominguez-Bello et al., 2010; Chu et al., 2017; Gomez et al., 2017). That is, maternal microbiomes could vertical and horizontal transmission to neonates and impact the composition of the microbiomes in neonates. For example, the vaginal bacteria are enriched in the microbiomes of vaginally delivered neonates and the skin microbiota are enriched in the microbiomes of the caesarean section delivered neonates (Dominguez-Bello et al., 2010, 2016).While, the difference of the microbiome induced by the delivery mode could be recovered with age (Chu et al., 2017; Dzidic et al., 2018). Meanwhile, neonates often acquire horizontally transmitted microbes from a family member in which they are in close contact with (Song et al., 2013). Besides, there are significant differences in the oral microbiomes between never breastfed and breastfed neonates, and these differences could last up to 7 years (Dzidic et al., 2018; Butler et al., 2022; Davis et al., 2022). Also, an increased abundance of *Veillonella* is found in never breastfed neonates at 2 months when compared with breastfed neonates (Butler et al., 2022). As for the effects of antibiotics used at the delivery on microbiome, studies have found that *Proteobacteria* is enriched in the oral microbiome of the neonates exposed to antibiotics while *Streptococcaceae* dominates in the unexposed neonates (Gomez-Arango et al., 2017). But these studies have not observed the changes of the neonatal oral microbiomes across several time points in the first 4 days postpartum and they also have not analyzed the effects of maternal microbiomes on neonatal oral microbiome in the early life (Lif Holgerson et al., 2015; Dzidic et al., 2018; Kennedy et al., 2019). Moreover, early microbial community plays a major role in the development of the oral microbiome and is a source of pathogenic and protective microorganisms in throughout life (Lamont et al., 2018; Xiao et al., 2020). To this end, we recruited 15 mother-neonate pairs to investigate the dynamics of the oral microbiome in neonates and assess the associated impact of the maternal microbiomes.

## Materials and methods

### Inclusion and exclusion criteria

All the participants were recruited from Maternal and Child Hospital of Hubei Province. This study was approved by the ethics committee of this hospital.

Pregnant women who met the following criteria were included: (a) pregnant women aged older than 18 years old; (b) those at greater than 28 weeks of gestation; (c) those who were pregnant for the first time and had a singleton pregnancy; and (d) those who were able to cooperate during the whole study and signed the informed consent form.

Pregnant women were excluded if they met one of the following criteria: (a) were multiparous woman; (b) were multigravida; (c) suffered from bacterial vaginitis, colpitis mycotica, and cervical ectropion; (d) had a history of polycystic ovarian syndrome, uterine fibroids, endometriosis, and adenomyosis; (e) underwent intravaginal administration during pregnancy or 1 year before pregnancy; (f) had a history of urological and/or vaginal surgery; (g) had a history of the use of antibiotics and/or probiotics during pregnancy; (h) received supragingival scaling, scaling and root planning, exodontia, and oral appliance during pregnancy; (i) were infected with human immunodeficiency virus, hepatitis B virus, hepatitis C virus, *Mycobacterium tuberculosis*, and *Treponema pallidum*; (j) had a history of toxic shock syndrome, autoimmune disease, familial disease, and metabolic syndrome; and (k) had a history of radiation therapy and chemotherapy.

Only healthy neonates with a gestational age over 37 weeks and a weight greater than 2,500 g were included in our study. Moreover, neonates meeting at least one of the following criteria were excluded: (a) were preterm and had a low birth weight; (b) had asphyxia; (c) suffered from infectious diseases, such as neonatal septicemia, infectious pneumonia, and cytomegalovirus infection; and (d) had congenital malformations.

### Samples collection

Characteristics of population informatics and medical history were collected. Moreover, the length of gestation, delivery mode, date of birth, and weight and height of the neonates were also recorded.

**Figure 1:**
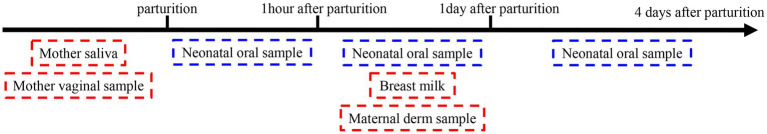
The sample timeline across the time points.

No drinks or food were consumed 2 h before collecting unstimulated whole saliva in the morning. For pregnant women, saliva was collected with sterile tubes before parturition. For neonates, sterile cotton swabs were used to collect saliva at three time points: the first hour after birth, the morning of the first day and the fourth day after birth. The maternal microbiomes of vagina were also collected with sterile cotton swabs before the parturition. Breast milk and the nipple derma microbiome were collected with sterile tubes on the first day after parturition (details showed in Figure 1). All samples were stored at −80°C for further analysis.

### Deoxyribonucleic acid extraction and PCR amplification

Deoxyribonucleic acid (DNA) from all samples was extracted with a HiPure Soil DNA Mini Kit (Magen, Shanghai, China) on a fume cupboard. After lysis, precipitation, and dissolution, the concentration of DNA was detected by a Nanodrop (Thermo Fisher Scientific, United States). Then, the same amount of DNA from all samples was diluted to 2 ng/μl for PCR amplification. Next, 16S ribosomal ribonucleic acid (rRNA) universal primer (27F/1492R) with the forward sequence 27F 5′-AGRGTTYGATYMTGGCTCAG-3′ and reverse sequence 1492R 5′- RGYTACCTTGTTACGACTT-3′ was used to amplify all the extracted DNA according to the instruction of the KAPA HiFi ReadyMix PCR Kit (Roche, United States). The process of PCR amplification was as follows: initial denaturation at 95°C for 3 min, 25 cycles including denaturation at 95°C for 30 s, annealing at 56°C for 30 s, extension at 72°C for 60 s, and final extension at 72°C for 5 min. Finally, a 1.5% agarose gel was used to detect whether the extracted DNA was of good quality. That is, the amplification products had a single band of 1.8 Kbp, which suggested that the DNA was of good quality.

### Second PCR amplification with barcode primers and sequencing

16S rRNA universal primer with Barcode was used to perform the second PCR amplification, and 2.0% agarose gel was used to detect whether these products had a single band. Then, these products were quantified with a Qubit (Thermo Fisher Scientific, United States) and mixed proportionally. After DNA damage repair, adapter ligation, and purification, the products were used to construct a SMRTbell library with a SMRTbell Template Prep Kit 1.0-SPv3 (PacBio, United States). Finally, the second PCR amplification products were sequenced with a DNA/Polymerase Binding Kit 3.0 (PacBio, United States) using the PacBio platform at Wuhan Frasergen Bioinformatics Co., Ltd. (Wuhan, China). The data were available in https://www.ncbi.nlm.nih.gov/sra/PRJNA892775.

### Sequence data pre-processing and analysis

In pre-processing, we used the Cutadapt software to remove primers and other types of unwanted adapter sequences, and to find and retain the sequences with 16S double-ended primers. Meanwhile, Usearch software were used to discard reads with lower quality and singleton, to merge or assemble pairs, to remove dereplication, to filter chimeric sequence, and to obtain sequences in good read quality and length. Then, the Usearch was selected to generate operational taxonomic units (OTUs), and 97% similarity of 16S sequences were typically constructed as an OTU. Next, all the OTUs were aligned and annotated with the Silva database to obtain bacteria at various taxonomic levels. Later, sequence data analysis was performed to further study the characteristics and differences in the microbiome in various groups.

A Venn diagram was used to describe the number of shared OTUs and unique OTUs among the groups and was visualized with the R software package. The Chao1 and Shannon indices were used to show the community richness and diversity of the microbiomes, respectively. These analyses were performed with Mothur version v.1.30. Three methods were selected to analyze the similarities and differences in the composition of the microbiome among individuals: principal coordinate analysis (PCoA), hierarchical clustering analysis, and nonmetric multidimensional scaling (NMDS). The bacterial community composition histogram was used to show the relative abundance of bacteria. However, only the relative abundance of species greater than 1% in each group was shown in the composition histogram. STAMP analysis was performed to analyze the differences between microbes, and ANOVA was selected to conduct this analysis among more than three groups. STAMP analysis was conducted with STMAP v2.0 software, and then the significant bacteria in this analysis were linearly fit. This figure was visualized by the R language ggplot 2 software package. Moreover, the relative abundances of the top 20 taxa at the species level for each group were shown in STAMP analysis figures. Furthermore, linear discriminant analysis effect size (LEfSe), which was a nonparametric factorial Kruskal–Wallis (KW) sum-rank test along with linear discriminant analysis (LDA), combined statistical significance and biological relevance, and was used to determine the biomarkers among groups. This analysis was performed on LEfSe software, and the relative abundances of the top 20 taxa at the species level of each group were shown in the LEfSe plot. A *P* value less than 0.05 was statistically significant, and the LDA threshold was 2.0.

## Results

There were 15 mothers and their neonates included in this study. The average reproductive age of the pregnant women was 28.3 years, and the average gestation time was 39 weeks and 6 days. There were 15 maternal oral microbiome samples (M group), 13 maternal nipple derma microbiome samples (D group), six maternal vaginal microbiome samples (V group), 13 maternal breast milk microbiome samples (Mi group), 12 neonatal oral microbiome samples within 1 h after birth (N1h group), 14 neonatal oral microbiome samples on the first day after birth (N1d group), and 15 neonatal oral microbiome samples on the fourth day after birth (N4d group). The details of included samples were showed in Table 1.

**Table 1:** The details of included samples.

	M^a^	Mi^b^	D^b^	V^a^	N1h^c^	N1d^c^	N4d^c^
001	√	√	√		√	√	√
002	√						√
003	√	√				√	√
004	√	√	√		√	√	√
006	√		√	√	√	√	√
010	√	√	√		√	√	√
011	√	√	√		√	√	√
013	√	√	√	√	√	√	√
014	√	√	√		√	√	√
017	√	√	√		√	√	√
018	√	√	√	√	√	√	√
019	√	√	√	√	√	√	√
020	√	√	√	√		√	√
023	√	√	√		√	√	√
024	√	√	√	√	√	√	√

M, maternal saliva; Mi, maternal breast milk; D, the nipple derma microbiome from mothers; V, maternal vaginal microbiome samples; N1h, neonatal oral microbiome samples within 1 h after birth; N1d, neonatal oral microbiome samples on the first day after birth; and N4d, neonatal oral microbiome samples on the fourth day after birth.

a samples were collected before parturition.

b samples were collected on the first day after parturition.

c samples were collected after parturition.

### Venn diagram of the microbiomes across multiple sites in neonates and mothers

**Figure 2:**
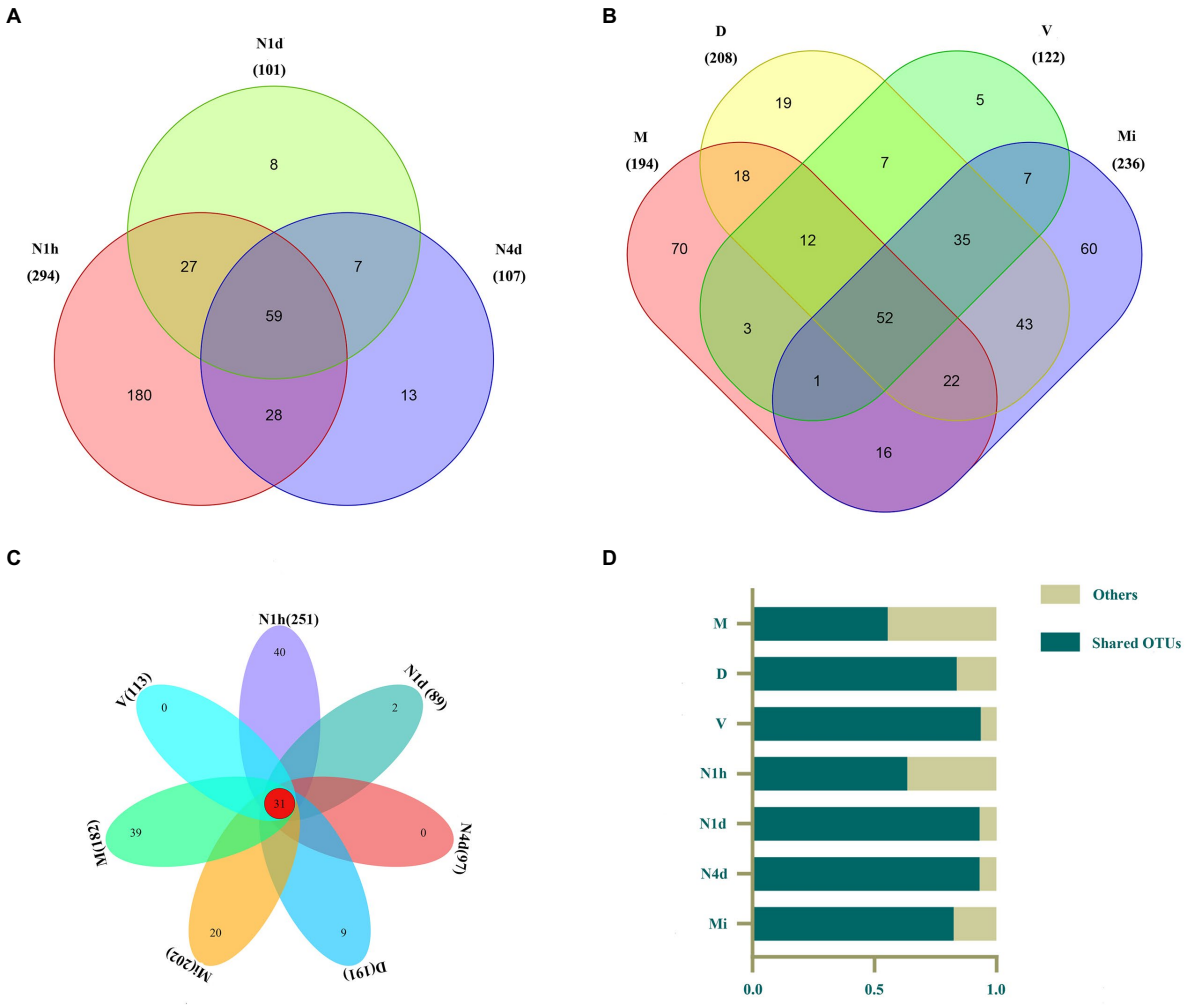
Venn diagram of the microbiomes across multiple sites in the neonates and their mothers. **(A–C)** The Venn diagram illustrated the number of OTUs in each group and showed the shared OTUs among the neonatal groups **(A)**, maternal groups **(B)**, and all seven groups **(C)**. **(D)** The contributions of these 31 shared OTUs to the microbiomes in all groups were shown in the histogram.

After clustering, 294, 101, 107, 194, 208, 122, and 236 OTUs were obtained in the N1h, N1d, N4d, M, D, V, and Mi groups, respectively (shown in Figures 2A,B). Interestingly, compared with the other groups, the N1h group had the greatest number of OTUs, followed closely by the Mi group. In addition, the number of OTUs in the neonates decreased rapidly within 4 days after birth, which showed that the number of bacteria in the neonatal groups decreased over time and that the composition of the oral microbiome in the neonatal groups obviously changed. Moreover, there were 59 OTUs shared by all the neonatal groups and 52 core OTUs found in all the maternal groups (shown in Figures 2A,B, and the shared OTUs shown in Supplementary Tables 1, 2). Notably, there were only 31 OTUs shared by both the neonatal groups and maternal groups, while over 50% of the microbiomes in all the groups were composed of the 31 core OTUs (shown in Figures 2C,D, and the shared OTUs shown in Supplementary Table 3).

### Alpha and beta diversity analysis of the microbiomes in the neonates and mothers

**Figure 3:**
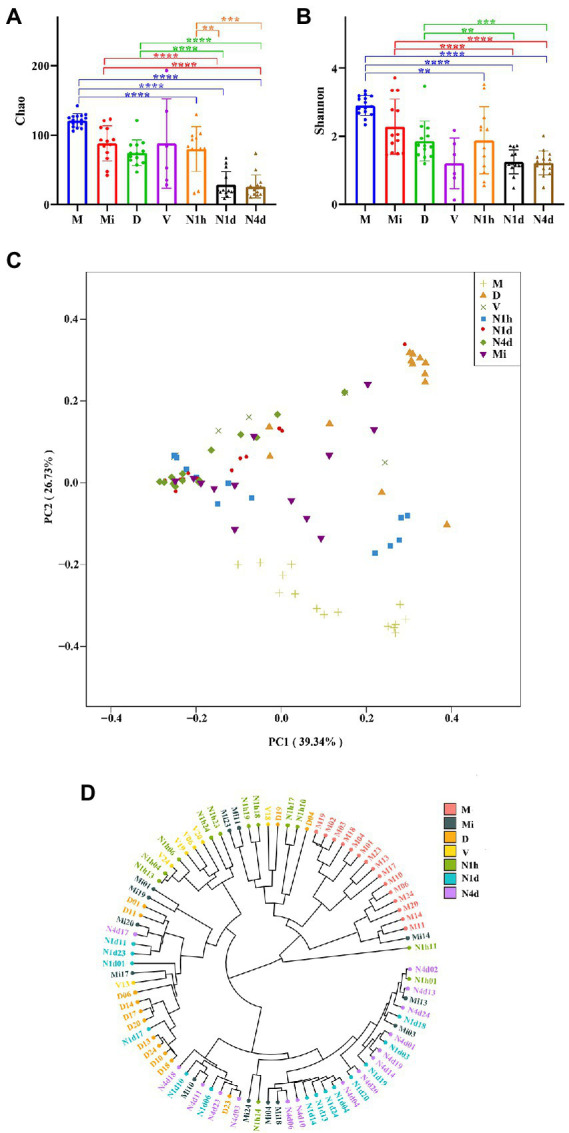
Alpha and beta diversity analysis of microbiomes in the neonates and their mothers. **(A,B)** The Chao1 index **(A)** and the Shannon **(B)** index of each group were analyzed by the number of OTUs in each group. Only the significance between maternal groups and neonatal groups were showed in the figures. ^****^p < 0.0001, ^***^p < 0.001, ^**^p < 0.01. **(C)** PCoA among all the groups was conducted on the basis of the weighted UniFrac distance and was shown along the first principal coordinate (PC1) and the second principal coordinate (PC2). PC1 and PC2 explained 39.34 and 25.73% of the variation, respectively. **(D)** The weighted UniFrac-Hierarchical clustering tree presented similarities and differences in evolution within all the samples. The distance between the branches represented the UniFrac distance, and the nodes of the tree represented the same UniFrac distance.

As shown in Figures 3A,B, the Chao1 and Shannon indices in the M group were higher than those in the neonatal groups, which suggested that the community richness and diversity in the M group were significantly higher and richer than those in the neonatal groups. Moreover, no significant difference in community richness and diversity was found between the N1h and Mi groups or between the V and D groups. Interestingly, the community richness of the oral microbiome significantly decreased over time after birth, while there was no difference in the community diversity among the three neonatal groups (shown in Figure 3B). Notably, the V group had the lowest Shannon index among all groups, indicating that there were fewest kinds of microbes in the vaginal microbiome than in the other microbiomes.

As shown in Figure 3C, the samples of the N1h group separated from those of the N1d group and N4d group in both axes. The samples of the M group were also far from all the neonatal groups on both axes. However, samples of the N1d group, N4d group, and Mi group could not be separated from each other at both axes. These PCoA results were also obtained in NMDS analysis (shown in Supplementary Figure 1). The hierarchical clustering tree showed that most samples from the N1d group and N4d group were clustered together and far from samples in the N1h group (as shown in Figure 3D). In addition, all the samples in the M group were clustered together and far from the samples in the neonatal groups. The samples in the Mi groups were not clustered together and near the samples in the N1d group and N4d group. These hierarchical clustering trees suggested that the composition of the oral microbiome in the N1h group was different from that in the N1d group and N4d group, and the composition in the M group was dramatically different from that in the neonatal groups. Moreover, the compositions of the microbiomes in the N1d group, N4d group, and Mi group were similar. All these results were in line with the results of PCoA and NMDS analysis.

### Composition analysis and LEfSe analysis of microbiomes

**Figure 4:**
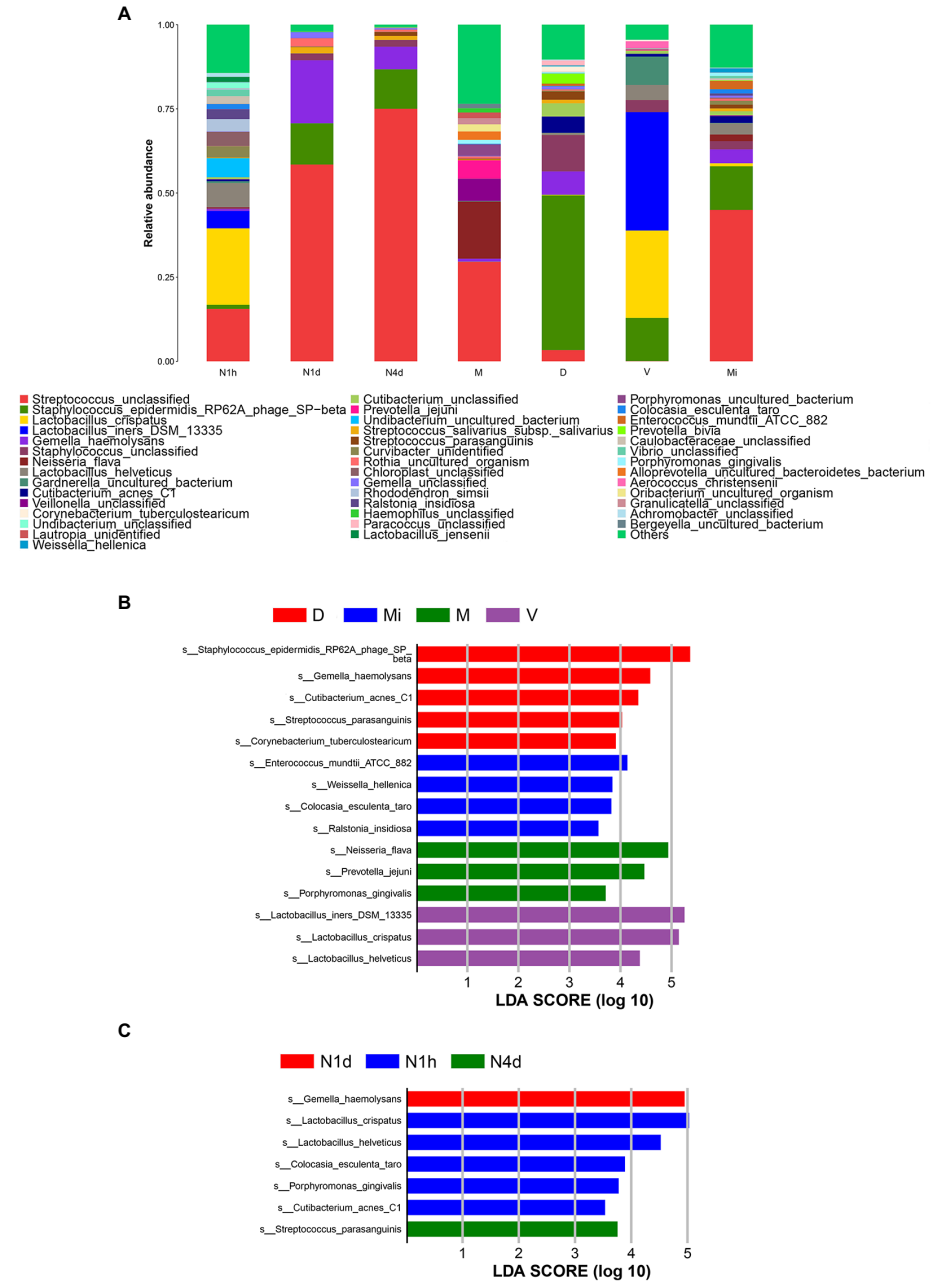
Composition analysis and LEfSe analysis of microbiomes. **(A)** The bacterial community composition histogram of each group was shown by the species whose relative abundance in each group was greater than 1%. **(B,C)** The LEfSe plot showed the differentially abundant bacteria in the maternal microbiomes **(B)** and the neonatal oral microbiomes **(C)**. The relative abundances of the top 20 taxa at the species level of each group were in the LEfSe plot. A *p* value less than 0.05 was considered statistically significant, and the LDA threshold was 2.0.

After alignment to the Silva database, there were 19 phyla, 26 classes, 108 families, 226 genus, and 375 species obtained in all samples. As shown in Figure 4A, *Streptococcus unclassified*, *Lactobacillus crispatus*, *Lactobacillus iners DSM 13335*, and *Lactobacillus helveticus* were the predominant bacteria in the N1h group. The dominant bacteria in the N1d group were similar to those in the N4d group and Mi group. That is, *Streptococcus unclassified*, *Gemella haemolysans*, and *Staphylococcus epidermidis RP62A phage SP-beta* were enriched in the three groups. Moreover, *Streptococcus unclassified*, *Neisseria flava*, and *Veillonella unclassified* were the main bacteria in the M group. The predominant bacteria in the V group were *Lactobacillus crispatus* and *Lactobacillus iners DSM 13335*. The relative abundance of *Staphylococcus epidermidis RP62A phage SP-beta* in the D group was over 40% and became the common bacterium.

Next, we performed LEfSe analysis to further search for statistical and biological biomarkers among the groups. When comparing the microbiomes from different maternal body sites, the biomarkers in the D group were *Staphylococcus epidermidis RP62A phage SP-beta*, *Cutibacterium acnes C1*, *Gemella haemolysans*, and *Streptococcus parasanguinis*. Moreover, *Ralstonia insidiosa* and *Weissella hellenica* were the biomarkers in the Mi group. In addition, *Lactobacillus iners DSM 13335*, *Lactobacillus helveticus*, and *Lactobacillus crispatus* were biomarkers of the vaginal microbiome (V group). *Neisseria flava*, and *Porphyromonas gingivalis* were the biomarkers in the M group (shown in Figure 4B). As shown in Figure 4C, the biomarkers found in the V group and D group also became biomarkers in the N1h group, such as *Lactobacillus crispatus*, *Lactobacillus helveticus*, and *Cutibacterium acnes C1*. *Gemella haemolysans* and *Streptococcus parasanguinis*, which were the biomarkers in the D group, were the biomarkers of the oral microbiome in the N1d group and N4d group, respectively. The biomarkers of the different maternal sites could also be biomarkers in the oral cavity of neonates, which suggested that the maternal environment contributed to the oral microbiome in neonates. Moreover, the changes in biomarkers in the neonatal groups once again showed that the oral microbiome was not stable during the 4 days after birth. Interestingly, cariogenic bacteria and periodontal pathogens colonized the oral cavity in the first hour after birth and even became biomarkers of the oral microbiome in the N1h group, such as *Rothia dentocariosa* and *Porphyromonas gingivalis* (shown in Supplementary Table 4).

### STAMP analysis of microbiomes to investigate different bacteria among groups

**Figure 5:**
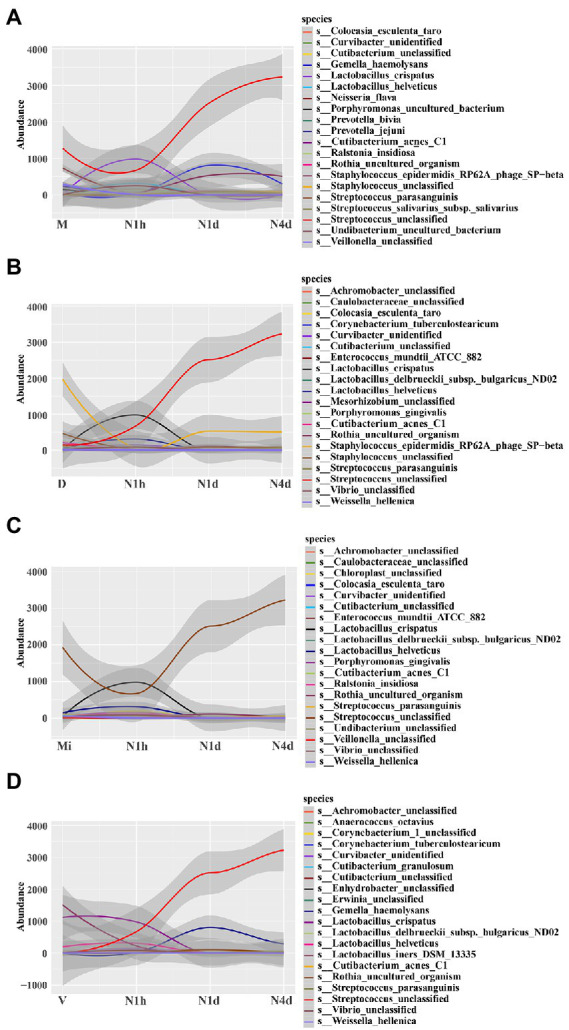
STAMP analysis of microbiomes to investigate different bacteria among groups. **(A–D)** STAMP analysis of neonatal groups in comparison with the M group **(A)**, D group **(B)**, Mi group **(C)**, and V group **(D)**, and then all the significant bacteria in each comparison were fit linearly. The *y*-coordinate represented the relative abundance of the species, and the *x*-coordinate represented the name of sample groups. The relative abundances of the top 20 taxa at the species level of each group were shown.

STAMP analysis was selected to identify different microbes between groups. When compared with the M group, the relative abundance of *Streptococcus unclassified* (details shown in Supplementary Figure 2A) and *Streptococcus parasanguinis* significantly decreased in the N1h group and sharply increased in the oral microbiome over time after birth, while the relative abundance of *Cutibacterium acnes C1*, *Lactobacillus crispatus*, and *Lactobacillus helveticus* obviously increased in the N1h group and then decreased in the N1d group and N4d group (shown in Figure 5A and Supplementary Figures 2A–E). Moreover, *Staphylococcus epidermidis RP62A phage SP−beta* increased rapidly after birth as time progressed, and the change in *Neisseria flava* was the opposite (details shown in Supplementary Figures 2F, G). Fascinatingly, *Veillonella unclassified* significantly decreased in the neonatal groups, and its relative abundance in the N1d group was the lowest among the four groups (details shown in Supplementary Figure 2H). Furthermore, *Gemella haemolysans* and *Rothia uncultured organism* increased transiently in the N1d group among the four groups (details shown in Supplementary Figures 2I, J). Among the comparisons, except for the common oral bacteria, many predominant bacteria of the vagina, skin and milk changed obviously in the neonatal groups, which also suggested that the composition of the oral microbiome in the neonatal groups was different from that in the maternal groups.

Further analysis showed that the relative abundance of *Staphylococcus epidermidis RP62A phage SP-beta* and *Staphylococcus unclassified*, the common skin bacteria in the D group, significantly increased in the N1d group and N4d group but was still lower than that in the D group (shown in Figure 5B and in Supplementary Figures 3A,B). For the other common skin bacterium, *Cutibacterium acnes C1* obviously decreased in the neonatal groups over time (shown in Figure 5B and in Supplementary Figure 3C). In the comparison with the Mi group, the relative abundance of *Streptococcus unclassified* in the N1d group and N4d group were higher (shown in Figure 5C and Supplementary Figure 3D). As shown in Figure 5D, the relative abundance of *Lactobacillus crispatus* and *Lactobacillus iners DSM 13335* (details shown in Supplementary Figures 3E,F), the main bacteria in the V group, sharply decreased in the neonatal groups after birth. These results showed that the time of the changes in these predominant bacteria was in line with the time when neonates came into contact with their mothers and also suggested that many common bacteria in other parts of the body would colonize the oral cavity as neonates came into contact with their mothers.

## Discussion

Our study first showed that the oral microbiome changed sharply during the 4 days after birth, and the characteristics of the oral microbiome in the neonates varied from those of their mothers. Moreover, the dynamics of the oral microbiome were in line with the changes in the maternal environment to which the neonates were exposed.

During parturition, the environment of newborns changed obviously and was exposed to the vagina, skin and environment. It was reasonable that the number of OTUs in the N1h group (only the babies in the N1h group were named the newborns in our study) was the highest among the groups. As the body site was the primary determinant of the community of the microbiome, many bacteria could only transiently survive in the oral cavity (Adler et al., 2021). For example, one study collecting the tongue microbiome of neonates from birth up to 3 days postpartum found that the composition of the tongue microbiome obviously changed and that many bacteria originating from the vagina, skin, and environment disappeared in the oral cavity over time (Ferretti et al., 2018). In our study, we also observed this phenomenon, and many bacteria, including *Prevotella bivia* and *Prevotella jejuni*, had disappeared in the N1d and N4d groups. This phenomenon could induce the diversity in the N1d group and N4d group lower than that in the N1h group. This phenomenon also resulted in that the bacterial communities in the N1h group were undifferentiated from those in the D group, V group and Mi group but could be essentially differentiated from those in the N1d group and N4d group. Apart from our study, a previous study found that the composition of the oral microbiome in newborns was similar to that of the skin microbiome as well as vaginal microbiome in their mothers (Dominguez-Bello et al., 2010). There was no significance of diversity analysis between the N1d group and N4d group, and the diversity in the two groups was significantly lower than that in the M group. All these results were in line with previous studies (Sulyanto et al., 2019; Williams et al., 2019; Lif Holgerson et al., 2020). That is, the study found that there was no significance in the Shannon index and Richness index in the neonatal oral microbiomes from day 2 to day 5 after birth (Williams et al., 2019). Moreover, some studies found that although the richness and diversity increased continuously with age in toddlers, they were still lower than those of the maternal group (Sulyanto et al., 2019; Lif Holgerson et al., 2020). In addition, we found that the composition of the oral microbiome in the N1d group and N4d group resembled that of the milk microbiome. As the bi-directional interaction between the mammary gland and the neonate’s oral cavity could provide a chance for the milk microbiome to settle in the oral cavity, the milk and neonatal oral microbiomes became increasingly similar to each other with age (Williams et al., 2019).

We also found that composition of maternal oral microbiome was different from that in neonatal oral microbiomes. Some reasons could be used to explain this phenomenon (Gomez and Nelson, 2017; Kaan et al., 2021; Yao et al., 2021). Firstly, the different dentition situation between maternal oral cavity and neonatal oral cavity. A study had showed that the tooth eruption affected the composition of the oral microbiome, and the relative abundance of *Streptococcus* was over 60% during the pre-dentate period while sharply decreased with the eruption of the primary incisors (Xu et al., 2022). Secondly, the huge age difference between mothers and neonates also induced the difference, and studies had found that some bacteria were less abundant at older ages (Huang et al., 2020; Schwartz et al., 2021). Thirdly, the varied diets between the mothers and neonates contributed to difference. For example, low carbohydrate high fat diet induced a decrease in the relative abundances of *Neisseria* and *Prevotella* spp. in adult, and breastfed induced an increased abundance of *Veillonella* in infants (Murtaza et al., 2019; Butler et al., 2022). Moreover, the different oral hygiene habits, including using floss and brushing, influenced the oral microbiome and using these habits could induce a low abundance of some caries-associated genus, such as *Campylobacter*, *Alloprevotella*, and *Leptotrichia* (Hallang et al., 2021; Rodrigues et al., 2021). Thus, it was reasonable that the composition of the oral microbiome in mothers was different from that in their neonates, and this was obtained in our study as well as in other studies (Dominguez-Bello et al., 2010; Ferretti et al., 2018; Williams et al., 2019).

As shown in STAMP analysis, the dynamics of the oral microbiome were in line with the changes in the environment, which was also obtained in other studies. For example, the oral cavity in newborns harbored vaginal bacteria when swabbed with gauze that was incubated in the maternal vagina 60 min before the caesarean section, and the oral microbiome community, diversity and richness significantly changed after the introduction of solid foods (Dominguez-Bello et al., 2016; Sulyanto et al., 2019). Interestingly, we found that there were many unique OTUs in different groups, but over 50% of the microbiomes in all the groups were composed of the 31 core OTUs. This result once again showed that although there was strong niche specialization in different sites of the human microbiome, there were still similarities in the composition of the microbiome (Human Microbiome Project C, 2012; Ding and Schloss, 2014). Moreover, the M group had the highest alpha diversities of OTUs, and the V group had the lowest alpha diversities, which was also in line with the findings of the human microbiome project (Human Microbiome Project C, 2012).

Many studies had shown that the mode of delivery affected the composition of the oral microbiome in newborns. That is, the oral microbiome in newborns delivered vaginally was similar in composition to the vaginal microbiome in their mothers; at the same time, the bacteria harbored in the oral cavity of the newborns delivered by caesarean section were similar to the bacteria on their mothers’ skin (especially referring to the abdomen; Dominguez-Bello et al., 2010; Chu et al., 2017). We found that the oral microbiome in the N1h group was generally dominated by the dominant bacteria of the vaginal microbiome. When we grouped the samples according to the mode of delivery, the dominant bacteria harbored in the oral cavity of the newborns were still vaginal bacteria (data not shown). The main reason might be that we could not determine whether the fetal membranes ruptured before the caesarean section or not. If the fetal membranes ruptured before the caesarean section, the vaginal microbiome would ascend and access the fetus (Wang et al., 2021). *Lactobacillus* was also a common genus in the endometrial microbiome and the fallopian (Chen et al., 2017). Once the fetal membranes ruptured, the fetus could access these microbiomes right away, and these bacteria could settle in the oral cavity. Moreover, one study detected 18 taxa in human fetal meconium by 11–14 weeks of gestation, and *Lactobacillus*, commonly housed in the vagina, was the most abundant genus among them (Rackaityte et al., 2020). If so, our results might be acceptable. The last reason was that we only studied the communities of the nipple derma microbiome, not the abdominal microbiome. It was well known that the composition of microbiome in various skin sites was different (Skowron et al., 2021; Ogai et al., 2022). Thus, we could not analyze the effect of the abdominal microbiome of the mothers on the oral microbiome of their newborns.

Though we are the first study to observe the dynamics of oral microbiomes in neonates during the first 4 days after birth, especially in first 1 h after birth, and analyze the effect of maternal microbiomes on the neonatal oral microbiomes in details. There were still some limitations of our study. First, we did not collect the details of fetal membrane rupture and the abdominal microbiome, which would amplify the impact of the vaginal microbiome on the neonatal oral microbiome as well as diminish the contribution of the skin microbiome to the neonatal oral microbiome. Second, as the gut microbiome from the mothers could colonize the neonatal oral cavity in the neonates, we might have made mistakes when analyzing the source of the oral microbiome in the neonates without collection data on the gut microbiome (Ferretti et al., 2018). Moreover, we could not analyze the contribution of the maternal oral microbiome to their baby’s initial bacterial community due to the limited follow-up time and the small sample size.

## Conclusion

In conclusion, we found that the oral microbiome in neonates was not stable during the 4 days after birth, and the maternal microbiomes of the vagina, skin, and milk could affect the composition of the oral microbiome in the neonates. These results aided us in developing a good understanding of the role of the maternal environment in the maturation of the neonatal oral microbiome.

## Data availability statement

The datasets presented in this study can be found in online repositories. The link of the datasets is shown in the article.

## Ethics statement

The studies involving human participants were reviewed and approved by Maternal and Child Hospital of Hubei Province. The participants provided their written informed consent to participate in this study.

## Author contributions

MD, HG, and JL designed the study. HG, JL, HY, and YL collected samples. YZ aided the recruitment of participants. HG and JL analyzed the data and wrote original manuscript. YJ, JZ, and MD revised the manuscript. All authors contributed to the article and approved the submitted version.

## Funding

This study was supported by the National Natural Science Foundation of China (No. 81771084).

## Conflict of interest

The authors declare that the research was conducted in the absence of any commercial or financial relationships that could be construed as a potential conflict of interest.

## Publisher’s note

All claims expressed in this article are solely those of the authors and do not necessarily represent those of their affiliated organizations, or those of the publisher, the editors and the reviewers. Any product that may be evaluated in this article, or claim that may be made by its manufacturer, is not guaranteed or endorsed by the publisher.
