# Supplementary material for: The dynamic communities of oral microbiome in neonates
Source: Front Microbiol. 2022 Dec 6;13:1052525. doi: 10.3389/fmicb.2022.1052525 (PMC9764626; doi:10.3389/fmicb.2022.1052525)
Supplement: Supplementary file 2 [file Data_Sheet_1.pdf]

Supplementary Material

Supplementary Figure 1

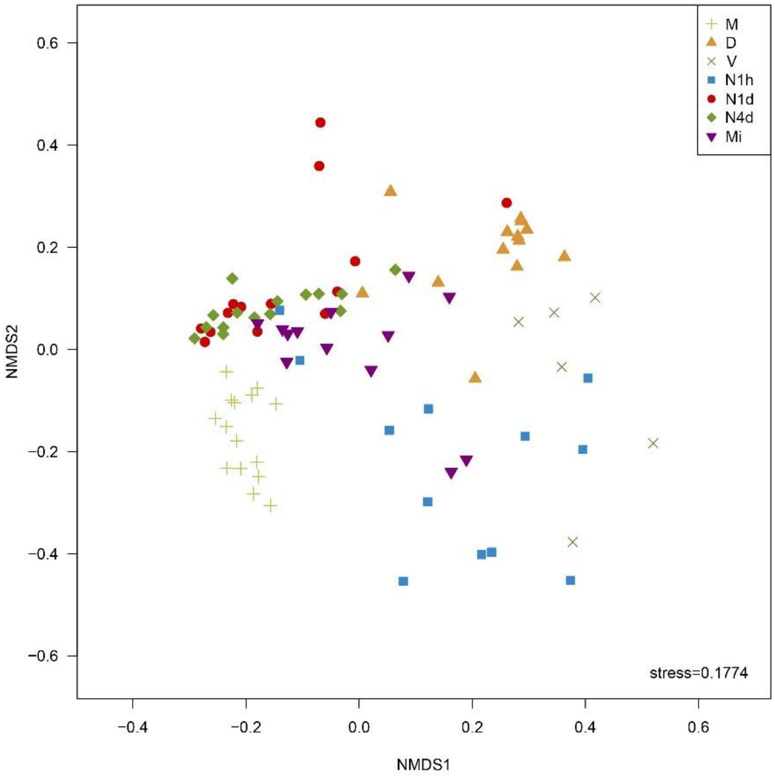

NMDS analysis of microbiomes in the neonates and their mothers. NMDS based on the Bray–Curtis distances illustrating the beta diversity of the microbiomes of all the groups. Each point represented a single sample.

Supplementary Figure 2

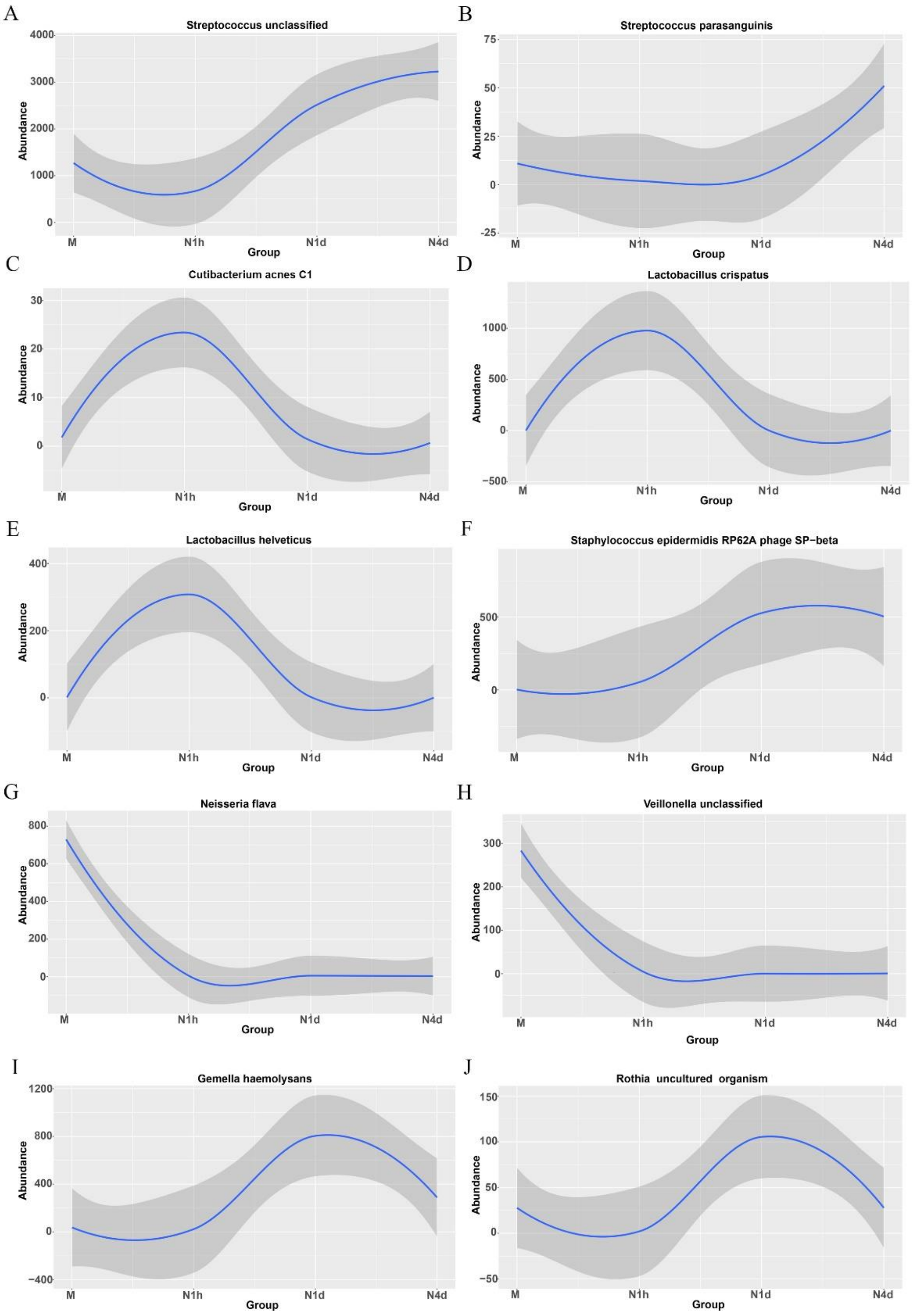

STAMP analysis of microbiomes to investigate different bacteria among maternal oral microbiome and neonatal oral microbiomes. STAMP analysis of neonatal groups in comparison with the M group, and then all the significant bacteria in each comparison were fit linearly**.** The y-coordinate represented the relative abundance of the species, and the x-coordinate represented the

name of sample groups. The relative abundances of the top 20 taxa at the species level of each group were shown.

Supplementary Figure 3

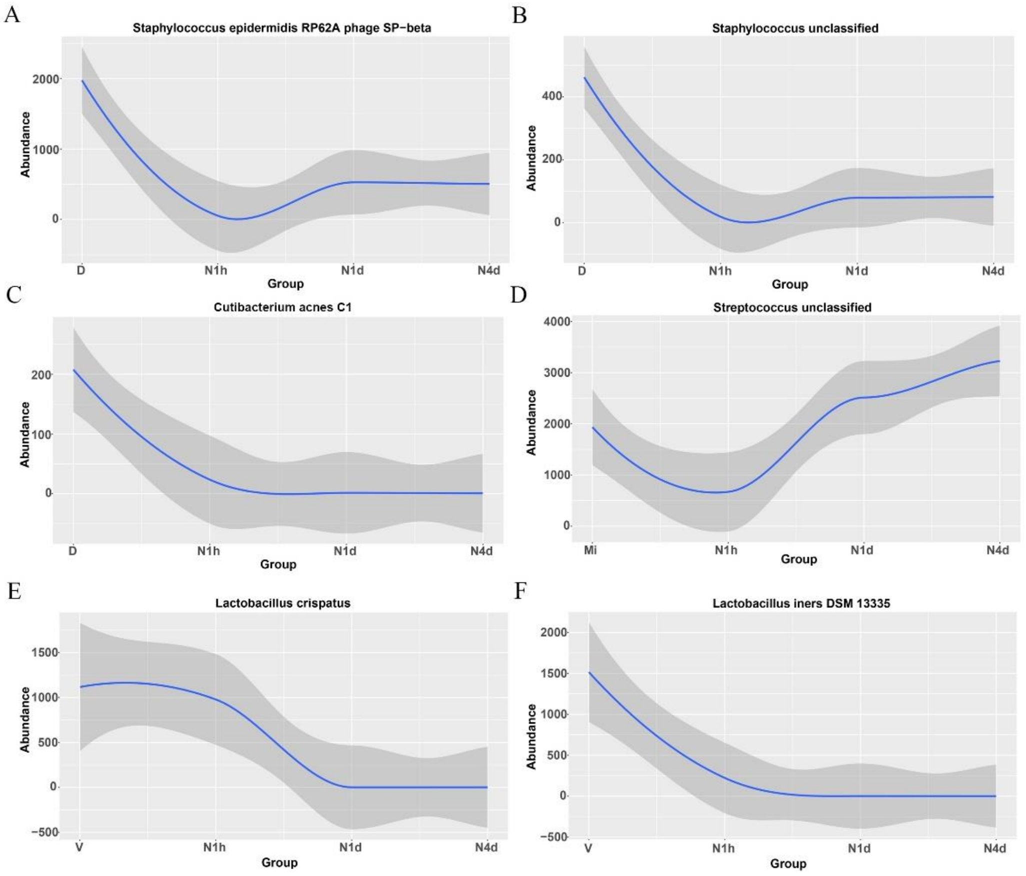

STAMP analysis of microbiomes to investigate different bacteria among maternal microbiomes and neonatal oral microbiomes. STAMP analysis of neonatal groups in comparison with D group (A, B and C), Mi group (D) and V group (E and F), and then all the significant bacteria in each comparison were fit linearly**.** The y-coordinate represented the relative abundance of the species, and the x-coordinate represented the name of sample groups. The relative abundances of the top 20 taxa at the species level of each group were shown.

Supplementary Table 1 The shared OTUs among the maternal samples

OTU ID	Taxonomy
OTU11	s_*Staphylococcus epidermidis RP62A phage SP_beta*
OTU13	g_*Cutibacterium*
OTU19	g_*Haemophilus*
OTU25	s_*Lactobacillus helveticus*
OTU26	s_*Aeromonas hydrophila YL17*
OTU41	s_*Lactobacillus iners DSM 13335*
OTU56	s_*Prevotella bivia*
OTU82	s_*Lonsdalea quercina subsp. populi*
OTU84	g_*Curvibacter*; s_*unidentified*

OTU85	g_*Gardnerella*; s_*uncultured bacterium*
OTU87	g_*Staphylococcus*
OTU96	s_*Capnocytophaga gingivalis*
OTU98	s_*Bifidobacterium breve*
OTU100	g_*Gemella*
OTU113	g_*Granulicatella*
OTU128	s_*Weissella hellenica*
OTU130	s_*Streptococcus salivarius subsp. salivarius*
OTU136	s_*Streptococcus parasanguinis*
OTU146	s_*Lactobacillus crispatus*
OTU147	s_*Bradyrhizobium denitrificans*
OTU148	s_*Kocuria marina*
OTU168	s_*Cutibacterium avidum 44067*
OTU182	s_*Neisseria oralis*
OTU187	g_*Vibrio*
OTU193	s_*Prevotella jejuni*
OTU207	g_*Escherichia_Shigella*
OTU209	s_*Porphyromonas gingivalis*
OTU210	g_*Rothia*; s_*uncultured organism*
OTU223	g_*Lactobacillus*
OTU229	s_*Pseudomonas aeruginosa VRFPA04*
OTU232	s_*Lactobacillus delbrueckii subsp. bulgaricus ND02*
OTU252	g_*Veillonella*
OTU255	s_*Cutibacterium acnes C1*
OTU262	s_*Atopobium vaginae DSM 15829*
OTU264	g_*Streptococcus*
OTU266	g_*Vibrio*
OTU275	s_*Brevundimonas vesicularis*
OTU277	s_*Bacteroides fragilis*
OTU290	s_*Gemella haemolysans*
OTU291	s_*Sphingomonas panni*
OTU292	g_*Prevotella 9*
OTU295	s_*Neisseria elongata subsp. glycolytica ATCC 29315*
OTU310	g_*norank*
OTU330	g_*Erwinia*
OTU343	s_*Micrococcus luteus*
OTU349	s_*Enterococcus mundtii ATCC 882*
OTU364	g_*Streptococcus*
OTU368	s_*Aerococcus christensenii*
OTU370	f_*Neisseriaceae*; g_*uncultured*; s_*uncultured organism*
OTU380	s_*Neisseria flava*
OTU385	s_*Staphylococcus epidermidis RP62A phage SP_beta*
OTU416	s_*Colocasia esculenta taro*

Supplementary Table 2 The shared OTUs among the neonate oral groups

OTU ID	Taxonomy
OTU1	f_*Caulobacteraceae;* g_*uncultured*
OTU11	s_*Staphylococcus epidermidis RP62A phage SP_beta*
OTU13	g_*Cutibacterium*
OTU19	g_*Haemophilus*
OTU25	s_*Lactobacillus helveticus*
OTU34	f_*Planococcaceae*
OTU41	s_*Lactobacillus iners DSM 13335*
OTU84	g_*Curvibacter*; s_*unidentified*
OTU85	g_*Gardnerella*; s_*uncultured bacterium*
OTU87	g_*Staphylococcus*
OTU98	s_*Bifidobacterium breve*
OTU100	g_*Gemella*
OTU107	g_*Pelomonas*; s_*uncultured bacterium*
OTU113	g_*Granulicatella*
OTU118	g_*Faecalibacterium*
OTU123	g_*Streptococcus*
OTU130	s_*Streptococcus salivarius subsp. salivarius*
OTU136	s_*Streptococcus parasanguinis*
OTU138	g_*Curvibacter*; s_*unidentified*
OTU141	g_*Parasutterella*; s_*uncultured organism*
OTU146	s_*Lactobacillus crispatus*
OTU149	g_*Streptococcus*
OTU178	g_*Mesorhizobium*
OTU179	g_*Bacteroides*
OTU198	g_*Stenotrophomonas*
OTU209	s_*Porphyromonas gingivalis*
OTU210	g_*Rothia*; s_*uncultured organism*
OTU219	g_*Streptococcus*
OTU226	g_*Bradyrhizobium*
OTU227	g_*Streptococcus*
OTU231	s_*Ralstonia insidiosa*
OTU232	s_*Lactobacillus delbrueckii subsp. bulgaricus ND02*
OTU240	g_*Undibacterium*; s_*uncultured bacterium*
OTU247	s_*uncultured Bosea sp.*
OTU252	g_*Veillonella*
OTU255	s_*Cutibacterium acnes C1*
OTU262	s_*Atopobium vaginae DSM 15829*
OTU264	g_*Streptococcus*
OTU277	s_*Bacteroides fragilis*
OTU287	g_*Bacillus*
OTU290	s_*Gemella haemolysans*
OTU292	g_*revotella 9*
OTU300	s_*Clostridium butyricum*
OTU304	g_*Bacteroides*

OTU310	g_*norank*
OTU327	s_*Bacillus] thermocloacae*
OTU330	g_*Erwinia*
OTU349	s_*Enterococcus mundtii ATCC 882*
OTU352	g_*Actinobacillus*
OTU360	s_*Corynebacterium suicordis*
OTU364	g_*Streptococcus*
OTU373	s_*Bacteroides dorei*
OTU380	s_*Neisseria flava*
OTU385	s_*Staphylococcus epidermidis RP62A phage SP_beta*
OTU387	s_*Algoriphagus sp. JJ018*
OTU395	s_*human gut metagenome*
OTU411	g_*Acinetobacter*
OTU414	g_*Lawsonella*; s_*uncultured organism*
OTU416	s_*Colocasia esculenta taro*

Supplementary Table 3 The shared OTUs among all groups

OUT ID	**Taxonomy**
OTU11	s_*Staphylococcus epidermidis RP62A phage SP_beta*
OTU13	g_*Cutibacterium*
OTU19	g_*Haemophilus*
OTU25	s_*Lactobacillus helveticus*
OTU41	s_*Lactobacillus iners DSM 13335*
OTU84	g_*Curvibacter*; s_*unidentified*
OTU85	g_*Gardnerella*; s_*uncultured bacterium*
OTU87	g_*Staphylococcus*
OTU98	s_*Bifidobacterium breve*
OTU100	g_*Gemella*
OTU113	g_*Granulicatella*
OTU130	s_*Streptococcus salivarius subsp. salivarius*
OTU136	s_*Streptococcus parasanguinis*
OTU146	s_*Lactobacillus crispatus*
OTU209	s_*Porphyromonas gingivalis*
OTU210	g_*Rothia*; s_*uncultured organism*
OTU232	s_*Lactobacillus delbrueckii subsp. bulgaricus ND02*
OTU252	g_*Veillonella*
OTU255	s_*Cutibacterium acnes C1*
OTU262	s_*Atopobium vaginae DSM 15829*
OTU264	g_*Streptococcus*
OTU277	s_*Bacteroides fragilis*
OTU290	s_*Gemella haemolysans*
OTU292	g_*Prevotella 9*
OTU310	g_*norank*
OTU330	g_*Erwinia*

OTU349	s_*Enterococcus mundtii ATCC 882*
OTU364	g_*Streptococcus*
OTU380	s_*Neisseria flava*
OTU385	s_*Staphylococcus epidermidis RP62A phage SP_beta*
OTU416	s_*Colocasia esculenta taro*
